# Air pollution and hospitalization of patients with idiopathic pulmonary fibrosis in Beijing: a time-series study

**DOI:** 10.1186/s12931-022-01998-8

**Published:** 2022-04-05

**Authors:** Lirong Liang, Yutong Cai, Baolei Lyu, Di Zhang, Shuilian Chu, Hang Jing, Kazem Rahimi, Zhaohui Tong

**Affiliations:** 1 Department of Clinical Epidemiology & Tobacco Dependence Treatment Research, Beijing Institute of Respiratory Medicine, Beijing Chaoyang Hospital, Capital Medical University, Beijing, China; 2 Centre for Environmental Health and Sustainability, Department of Health Sciences, University of Leicester, Leicester, LE1 7RH UK; 3 Huayun Sounding Meteorology Technology Corporation, Beijing, China; 4 Nuffield Department of Women’s & Reproductive Health, University of Oxford, Oxford, UK; 5 Department of Respiratory and Critical Care Medicine, Beijing Institute of Respiratory Medicine, Beijing Chaoyang Hospital, Capital Medical University, Beijing, 100020 China

**Keywords:** Air pollutants, Interstitial lung diseases, Disease progression, Developing countries, Particulate matter

## Abstract

**Background:**

A small number of studies suggested that air pollution was associated with idiopathic pulmonary fibrosis (IPF) exacerbation, incidence and mortality. However, no studies to date were conducted in regions where air pollution is substantial. We aimed to investigate whether there are associations between acute increases in air pollution and hospitalization of patients with a confirmed primary diagnosis of IPF in Beijing.

**Methods:**

Daily count of IPF hospitalizations (International Classification of Disease-10th Revision, J84.1) was obtained from an administrative database for 2013–2017 while daily city-wide average concentrations of PM_10_, PM_2.5_, NO_2_, Ozone, SO_2_ were obtained from 35 municipal monitoring stations for the same period. The association between daily IPF hospitalization and average concentration of each pollutant was analyzed with a generalized additive model estimating Poisson distribution.

**Results:**

Daily 24-h mean PM_2.5_ concentration during 2013–2017 was 76.7 μg/m^3^. The relative risk (RR) of IPF hospitalization per interquartile range (IQR) higher (72 μg/m^3^) in PM_2.5_ was 1.049 (95% CI 1.024–1.074) and 1.031 (95% CI 1.007–1.056) for lag0 and moving averages 0–1 days respectively. No significant associations were observed for other lags. Statistically significant positive associations were also observed at lag0 with SO_2_, Ozone and NO_2_ (in men only). Positive associations were seen at moving averages 0–30 days for PM_10_ (RR per 86 μg/m^3^: 1.021, 95% CI 0.994–1.049), NO_2_ (RR per 30 μg/m^3^: 1.029, 95% CI 0.999–1.060), and SO_2_ (RR per 15 μg/m^3^: 1.060 (95% CI 1.025–1.097), but not with PM_2.5_ or Ozone.

**Conclusions:**

Despite improvement in air quality since the implementation of clean air policy in 2013, acute exposure to higher levels of air pollution is significantly associated with IPF hospitalization in Beijing. Air quality policy should be continuously enforced to protect vulnerable IPF populations as well as the general public.

**Supplementary Information:**

The online version contains supplementary material available at 10.1186/s12931-022-01998-8.

## Background

Idiopathic pulmonary fibrosis (IPF) is the most common type of interstitial lung diseases (ILD), characterized by inflammation and fibrosis of the pulmonary parenchyma [1]. The exact causes of IPF are still largely unknown, with some studies suggesting that inhaled exposures such as cigarette smoke or wood dust may be among the triggers [2]. Patients with IPF experience progressive deterioration of lung function, impaired quality of life and an estimated median survival of three to five years after diagnosis [1, 3, 4]. An acute respiratory worsening could significantly alter the natural history of IPF, potentially resulting in hospitalization and mortality. However, an immediate cause cannot always be established for around half of the IPF acute worsening cases [5].

Unlike major respiratory conditions (e.g. chronic obstructive pulmonary disease (COPD), bronchitis, asthma), for which evidence of harmful effects from ambient air pollution is well documented [6–8], evidence for IPF morbidity and mortality remains relatively scarce. Increased exposure to Ozone (O_3_) were found to be associated with acute exacerbation of IPF six weeks after exposure in 436 and 192 patients in South Korea [5] and France [9] respectively, while number of hospitalizations was significantly higher following the days when levels of air pollutants including nitrogen dioxide (NO_2_), Particulate Matter with a diameter less than or equal to 10 μm (PM_10_) or 2.5 μm (PM_2.5_) were high in Santiago, Chile [10]. A more recent study of 152 patients in Japan concluded that exposure to PM_2.5_ and nitric oxide in the previous 30 days were significantly associated with IPF acute exacerbation [11]. Two studies in the United States found that higher air pollution exposure was associated with a lower level [12], or a faster decline [13], of forced vital capacity (FVC) among a small group of IPF patients. Another two US cohorts found that long-term exposure to air pollutants, in particularly those traffic-related (e.g. elemental carbon, nitrogen oxides) may increase the risk of interstitial lung abnormalities [14, 15]. Whilst these studies demonstrated that ambient air pollution could significantly impact IPF disease progression, three other studies from France [9], Italy [16] and South Korea [17] reported that higher NO_2_ and/or PM exposure was associated with IPF incidence and mortality. To date, no studies have been conducted in a region where ambient air pollution is substantial.

Air quality in Beijing has been progressively improving due to the implementation of the Clean Air Action Plan (CAAP) since 2013 [18], although average concentrations are still many times higher than the World Health Organisation (WHO) targets. In this ecological study, we compiled both air pollution and hospitalization data during 2013–2017 (i.e. the first phase of CAAP), and ran time-series analyses to investigate the acute effects of ambient air pollution on IPF hospitalization risk in Beijing. We additionally investigated whether the associations differed by patients’ characteristics, including age, sex and respiratory co-morbidity (COPD and asthma), as well as by seasonality.

## Methods

### Air quality data

Since 2013, major air pollutants have been routinely measured at 35 monitoring stations across Beijing, following the new Chinese national standard (GB 3095-2012). These monitoring stations were set up at locations that best represent different sources, for example, sources from road vehicles, urban anthropogenic activities, rural background and wider regional transport or background sources (Additional file 1: Fig. S1). At each station, a daily 24-h mean concentration for each pollutant except O_3_ is reported based on hourly data that are available for at least 20 h in every 24 h. For O_3_, a daily maximum 8-h moving mean concentration is reported based on hourly data that are available for at least 6 h in every 8 h.

Daily city-wide mean concentrations of PM_10_, PM_2.5_, NO_2_, SO_2_ (sulphur dioxide), and O_3_ were obtained from Jan 18th, 2013 to Dec 31st, 2017 (1809 days) by averaging daily mean readings from all 35 stations. Of the 1809 days, five days with missing data on all air pollutants were excluded. In addition, for O_3_, daily mean concentration was missing for another 34 days. As a result, the analyses of PM_10_, PM_2.5_, NO_2_ and SO_2_ were based on the 1804-day dataset whilst the analysis of O_3_ were based on a 1770-day dataset. Daily mean temperature (°C) and relative humidity (%) were provided by the Beijing Meteorological Service website.

PM_2.5_, as a general air quality indicator, was not routinely monitored in Beijing before 2013. In order to examine whether there is difference on the association between PM_2.5_ and IPF hospitalization before and after the CAAP policy, we further obtained daily mean concentration of PM_2.5_ data during Apr 1st, 2008 and Dec 31st, 2012 (1508 days) from a single monitor operated by the US Embassy in Beijing. It was previously reported that within a radius of 40 km, this monitor covers 79.2% of the total population, 97.8% tertiary hospital and 79.3% secondary hospitals in Beijing that are eligible to admit IPF cases [19]. The PM_2.5_ data from this single monitor have been widely used in previous studies [19, 20]. To further verify the observations from this single PM_2.5_ monitor, we correlated the daily mean concentrations of this monitor with a nearby 1.5 km-apart government monitoring station using data from 2013. Overall, observations from the two monitors showed a high correlation (r = 0.93).

### IPF hospitalization data

Daily counts of IPF hospitalization for the periods 2008–2012 and 2013–2017 were obtained from a database compiled by the Beijing Public Health Information Center. In Beijing, every government or private hospital at secondary or tertiary level is eligible to provide specialised care and required to submit the front page of each electronic hospital discharge record to the database [18]. Detailed information including age, sex, residential address (at urban district or township level), admitted hospital, date of admission, primary discharge diagnosis and the corresponding International Classification of Diseases, 10th Revision (ICD-10) code was recorded. All hospitalization records for patients aged ≥ 18 years, resided in Beijing on a permanent basis (i.e. > 6 months), and with a primary discharge diagnosis of IPF (ICD-10: J84.1) were extracted for this analysis. In Beijing, IPF patients could either self-refer to the outpatient department or be referred from primary care, and hospitalization will be warranted if their symptoms were found to be deteriorated. The primary diagnosis of IPF at discharge was coded by clinicians after reviewing the patient’s medical record (i.e. diagnostic terminology [10]) during hospitalization. All IPF admissions were from a total of 141 hospitals (75 tertiary and 66 secondary). Data were de-identified and it is not possible to identify individual patients, therefore informed consent was not required from this secondary data analysis. This study was approved by the Research Ethics Board of Beijing Chaoyang Hospital (approval number 2018-ke-303).

### Statistical analyses

We defined same-day exposure as lag0 (e.g. the admission date) and examined a priori single-day exposure up to five days (single-day lag0 to lag4) and exposure of moving averages 0–1, 0–2, 0–3, and 0–4 before admission date.

While our primary analysis aim was to determine the acute effects of air pollution on IPF hospitalization, we additionally investigated the moving averages 0–30 period to account for potential subacute effects of air pollution on development of worsening symptoms of IPF. The exact exposure time window during which IPF patients develop an episode of worsening symptoms remains debated, although by convention the onset should be within one month prior to diagnosis [21]. Exposure time windows of some recent studies on confirmed IPF acute exacerbation cases were generally between the preceding 0–42 days [5, 11].

The associations between daily IPF hospitalization and average concentration of each pollutant were analyzed with a generalized additive model (GAM) estimating Poisson distribution:

$${\text{Log}}\left[ {{\mathbf{E}}({\mathbf{Y}}_{{\mathbf{t}}} )} \right] = intercept + \beta C - {\mathbf{i}}_{{\mathbf{t}}} + {\text{ps}}\left( {calendar \, time_{t} ,{ 9}} \right) + {\text{ps }}\left( {temp_{t} ;{ 3}} \right) + {\text{ps }}\left( {RH_{t} ,{ 3}} \right) + public holiday_{t} + day \, of \, week_{t}$$

where **E(Y**_**t**_**)** represents the number of IPF cases on day t; ‘**C**’ is the city-averaged concentration of the pollutant and **i** is the day lag; **β** represents log-relative risk (RR) of IPF hospitalisation associated with a unit increase in the pollutant mean concentration; **ps** indicates penalised spline function to filter out long-term trends and seasonal patterns in daily IPF hospitalisations. **temp** and **RH** are the daily mean temperature (°C) and relative humidity (%) respectively at lag0; public holiday and day of week were included as categorical variables. Degrees of freedom (df) for calendar time, temperature and relative humidity were selected based on the parameters used in previous studies [22–24].

Each association was investigated in two-pollutant models if Spearman correlation ratios between these pollutants were less than 0.7. Subgroup analyses were conducted by age (18–64 years vs. ≥ 65 years), sex, season (warm vs. cool), asthma (yes vs. no) and COPD (yes vs. no). We conducted the sub-group analyses at lag0 only because for most studied pollutants, the association was found to be much stronger at lag0 in the main model. The Z-test was used to compare the two estimates derived from each sub-group.

The smoothing function of the generalized additive model was used to graphically analyse the exposure–response relationships between the log-RR of IPF hospitalization and air pollutant concentrations at lag0.

We did several sensitivity analyses by altering the generalized additive model: (1) to exclude calendar time, as long-term trends and seasonal patterns might also be partly related to pollutant concentrations; (2) to replace calendar time with an interaction term of exposure-by-season; (3) to increase the degrees of freedom of temperature and humidity to six; and (4) to model moving averages 0–4 of temperature and humidity instead of the current day (lag0). The latter two sensitivity analyses were to adjust potential non-linear and lagged confounding effects of weather conditions.

All statistical analyses were conducted in R (version 3.0.2) using packages MGCV, DPLYR, and TTR. Relative risk (RR) of hospitalisation for IPF per interquartile range (IQR) increase for each air pollutant were calculated and presented.

## Results

Across Beijing, daily 24-h mean PM_2.5_ concentration during 2008–2012 and 2013–2017 was 88.9 and 76.7 μg/m^3^ respectively (Table 1), as compared to the recently updated WHO guideline of 15 μg/m^3^. For NO_2_ and SO_2_, daily 24-h mean concentration was 50.5 μg/m^3^ and 15.1 μg/m^3^ respectively; whilst daily 8-h mean concentration for O_3_ was 95.8 μg/m^3^. Spearman correlations between each pair of pollutants were low to moderate (i.e. r < 0.7), except the high correlation between PM_10_ and PM_2.5_ (r = 0.93), PM_10_ and NO_2_ (r = 0.73), PM_2.5_ and NO_2_ (r = 0.78) (Additional file 1: Table S1). In 2013–17, 11,974 IPF admissions were recorded in Beijing, with a daily mean of six (Table 1). Number of admissions was higher among patients aged ≥ 65 years, and patients without COPD, but no major differences were seen between sexes or seasons.

**Table 1:** Daily air pollutant concentrations and IPF hospitalizations in Beijing, 2013–2017

	Minimum	Maximum	Mean (SD)	Median (IQR)
Air pollutant concentrations (μg/m^3^)				
PM_10_	10.0	820.0	109.7 (79.1)	91.0 (54.0–140.0)
PM_2.5_ (2013–2017)	5.0	467.0	76.7 (66.7)	58.0 (29.0–101.0)
PM_2.5_ (2008–2012)	3.0	552.5	88.9 (73.7)	69.0 (36.0–118.9)
NO_2_	8.0	155.0	50.5 (24.2)	44.0 (33.0–63.0)
SO_2_	2.0	139.0	15.1 (18.4)	8.0 (4.0–19.0)
O_3_	2.0	292.0	95.8 (62.2)	83.0 (50.0–135.0)
O_3_, warm season	4.0	292.0	128.5(63.5)	126.0 (78.0–175.0)
O_3_, cool season	2.0	233.0	61.5 (37.4)	59.0 (35.0–84.0)
IPF hospitalization (number of cases per day), 2013–2017 (n = 11,974)				
Total	0	22	6.4 (3.9)	6.0 (3.0–9.0)
Male	0	14	3.6 (2.5)	3.0 (2.0–5.0)
Female	0	12	2.8 (2.1)	2.0 (1.0–4.0)
Age < 65 years	0	12	1.9 (1.8)	2.0 (1.0–3.0)
Age ≥ 65 years	0	17	4.5 (2.9)	4.0 (2.0–6.0)
Warm season, May to October	0	22	6.4 (3.8)	6.0 (3.0–9.0)
Cool season, November to April	0	21	6.5 (4.0)	6.0 (3.0–9.0)
With COPD*	0	4	0.4 (0.7)	0.0 (0.0–1.0)
Without COPD	0	21	6.0 (3.7)	6.0 (3.0–9.0)
With asthma	0	2	0.1 (0.3)	0.0 (0.0–0.0)
Without asthma	0	21	6.4 (3.9)	6.0 (3.0–9.0)

^*^COPD chronic obstructive pulmonary disease

**Fig. 1:**
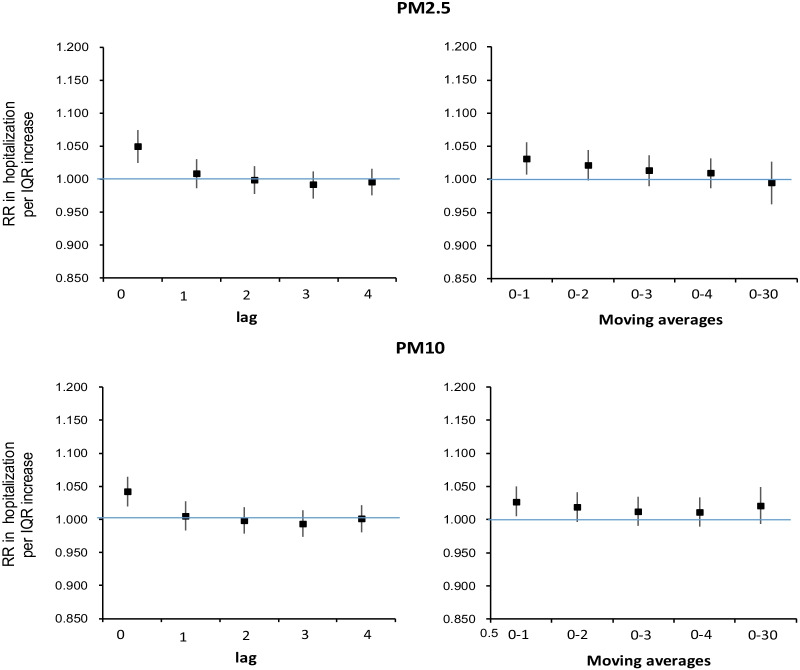
Associations between per IQR higher in PM_2.5_ and PM_10_ at different time lags and daily hospital admissions risk for IPF in Beijing during 2013–2017

Acute exposure to higher PM_2.5_ exposure during 2013–17 was significantly associated with IPF hospitalization in a relatively short lag (Fig. 1, Additional file 1: Table S2). The RR per IQR (72 μg/m^3^) higher was 1.049 (95% CI 1.024–1.074) at lag0, 1.031 (95% CI 1.007–1.056) at moving averages 0–1 and 1.021 (95% CI 0.998–1.044) at moving averages 0–2. Expressed per 10 μg/m^3^ higher of PM_2.5_, the RRs are 1.007 (95% CI 1.003–1.010), 1.004 (95% CI 1.001–1.008) and 1.003 (95% CI 0.999–1.006) respectively. Results of PM_10_ were similar to those of PM_2.5_ except that for the moving averages 0–30, where there was a positive association with PM_10_ (RR: 1.021, 95% CI 0.994–1.049), compared to a null association with PM_2.5_ (RR: 0.994, 95% CI 0.962–1.027).

During 2008–2012, the RR of hospitalization for IPF at lag0 per interquartile range (IQR) (83 μg/m^3^) higher in PM_2.5_ was 1.062 (95% CI 1.025–1.101) (Additional file 1: Fig. S2, Additional file 1: Table S3). The RR was reduced at lag1, lag2 and lag3 but remained positive. Positive associations with consistent RR estimates were observed for moving averages 0–1 up to 0–4. For example, the RR at moving averages 0–1 per IQR higher in PM_2.5_ was 1.054 (95% CI 1.020–1.090).

**Fig. 2:**
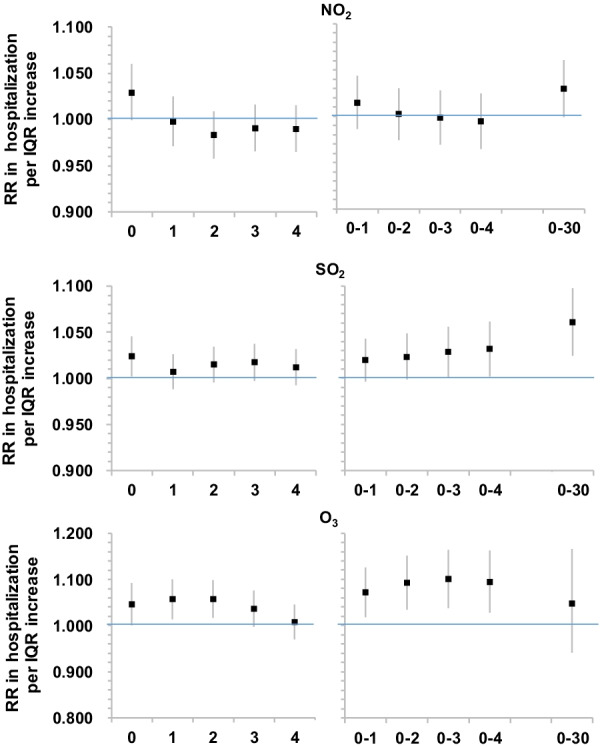
Associations between per IQR higher in gaseous pollutants at different time lags and daily hospital admissions risk for IPF in Beijing during 2013–2017

Associations varied by gaseous pollutants in different lags (Fig. 2, Additional file 1: Table S4). A marginally positive association was observed for each IQR (30 μg/m^3^) higher of NO_2_ on both lag0 and moving averages 0–30 (RR: 1.029, 95% CI 0.999–1.060). A significant positive association was observed for SO_2_ at lag0 only (RR: 1.023 (95% CI 1.002–1.045) per IQR (15 μg/m^3^) higher). Positive effect estimates of SO_2_ appeared to be in an increasing trend. For example, the RR at moving averages 0–1 and 0–30 was 1.019 (95% CI 0.996–1.043) and 1.060 (95% CI 1.025–1.097) respectively. A significant positive association was seen at lag0 for O_3_ (RR: 1.045, 95% CI 1.000–1.092 per IQR (85 μg/m^3^) higher). Similar associations were observed at least until lag3. Significant positive associations were seen for moving averages 0–1 up to 0–4 (RR: 1.093, 95% CI 1.028–1.162); however, the association at moving averages 0–30 was positive but not statistically significant (RR: 1.047, 95% CI 0.941–1.165). The associations with O_3_ were statistically significant during cool season but not in warm season (Additional file 1: Table S5).

The main results remained stable in all the sensitivity analyses (Additional file 1: Table S6) and two-pollutant models (Additional file 1: Table S7). During 2013–2017, positive exposure–response curves at lag0 were observed at least up to 300 μg/m^3^ for PM_2·5_, whereas various patterns were observed for the gaseous pollutants (Additional file 1: Fig. S3).

**Fig. 3:**
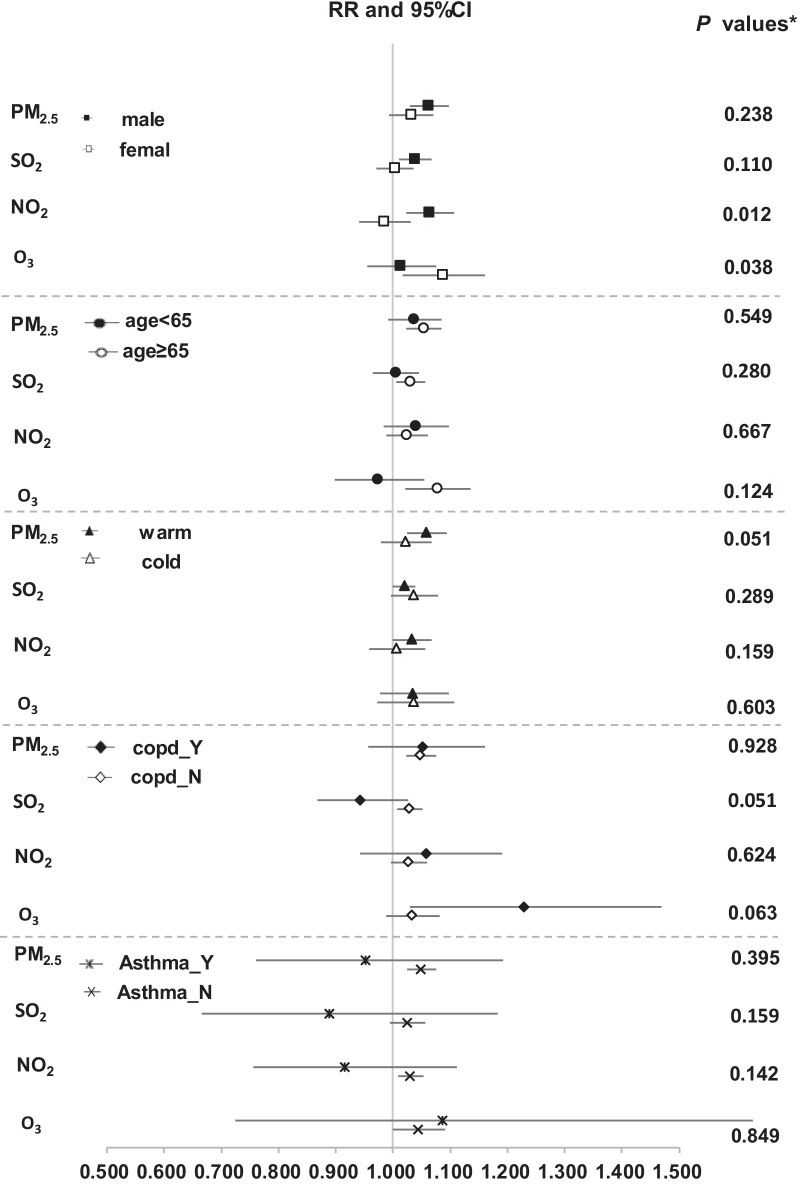
Subgroup analyses on the associations between each pollutant (per IQR higher) and hospitalization for IPF in single-pollutant models at Lag0

Stronger positive associations with all pollutants except O_3_ were seen in men as compared to women, with the interaction term with NO_2_ reaching statistical significance (Fig. 3). In men, per IQR higher of exposure to NO_2_ at lag0 was significantly associated with IPF hospitalization (RR: 1.064, 95% CI 1.023–1.107). While in women, per IQR higher exposure to O_3_ at lag0 was significantly associated with IPF hospitalization (RR: 1.087, 95% CI 1.017–1.161). There were no significant effect modifications by age, season, COPD or asthma with each pollutant, although there is a tendency that patients aged ≥ 65 years had a higher hospitalization risk with higher exposure to each pollutant except NO_2_. For IPF patients with COPD, hospitalization risk (RR: 1.230, 95% CI 1.030–1.469) was significantly higher as per IQR higher O_3_ exposure, as compared to patients without COPD (RR: 1.034, 95% CI 0.988–1.082).

## Discussion

Our findings suggest that acute exposure to higher levels of air pollutants, both particulates and gases, was significantly associated with IPF hospitalization in Beijing during 2013–2017. The magnitude of the associated risk is comparable to what was previously observed in Beijing for COPD exacerbation requiring hospitalization [18].

To date, only four studies reported associations between short-term air pollution exposure and acute exacerbation or hospitalization of IPF [5, 9–11], with mixed findings observed across the studies. This may be due to the differences in exposure levels and toxicity, study populations in different climate zones and ethnic groups, analytical approaches as well as the criteria in diagnosing and/or admitting IPF patients.

Our finding of an association between acute PM_2.5_ exposure and IPF hospitalization is an important addition to current knowledge. Previously, the Korean study did not have PM_2.5_ data [5], whilst the French study did not find an association between short-term PM_2.5_ and acute exacerbation of IPF among 192 patients [9]. The time-series study in Santiago, Chile reported that PM_2.5_ exposure in the previous four days was a significant risk factor of IPF hospitalization [10], which is in line with our study. Despite the concentrations of ambient PM_2.5_ was much higher in Beijing (77 μg/m^3^ 2013–2017) than in Santiago (29 μg/m^3^ 2001–2012), the risk estimate in our study was relatively smaller. This may reflect differences in statistical approaches used (e.g. the Chilean study ran separate region-specific time-series analyses and then pooled all the estimates together), PM_2.5_ toxicity and population characteristics.

Our study has suggested that more acute exposure (i.e. past three days) to PM_2.5_, as compared to more recent exposure (i.e. past 30 days), was relatively more important in leading to IPF hospitalization. This is in contrast to a Japanese study of 152 patients in which a significant positive association between IPF acute exacerbation and prior 30-day average PM_2.5_ exposure was found [11]. We did find, however, a positive association between prior 30-day average PM_10_ exposure and IPF hospitalization risk. This finding highlighted the potential of coarse PM in triggering symptoms among IPF patients via direct deposition in the respiratory tract over a sub-acute period [24]. In Beijing, studies have shown that sources of PM_10_ mainly come from dust whilst for PM_2.5_, traffic is the largest source with coal combustion and biomass burning contributing in winter months [25, 26]. We suspect that the differences in sources as well as compositions may have influenced the time course of both coarse PM and fine PM on IPF hospitalization. More research is needed to confirm this, especially in cities where sources of air pollution are diverse. Numerous mechanistic studies have indicated that PM could cause oxidative stress via over-production of reactive oxygen species [2, 24], which eventually leads to cellular damage and shortened telomeres, a recognized risk factor of IPF [27]. Both coarse and fine PM could also induce local and systemic inflammation of similar magnitude [28]. Collectively, these biological pathways could accelerate the lung injury and fibrotic process among the IPF patients.

We found that, as compared to the associations with PM_2.5_ observed in 2013–2017, the associations observed in 2008–2012 were relatively stronger and the lag time to hospitalization was longer (5 vs. 2 days). It should be noted that PM_2.5_ data from 2008 to 2012 were only based on a single monitor. Although this is not a direct assessment of health benefits resulting from the Clean Air Action Plan, our findings seem to echo previous reports that improvement in air quality has indeed improved several morbidity and mortality outcomes in the Chinese population [18, 29, 30].

This study found that acute exposure to O_3_ up to preceding five days was significantly associated with IPF hospitalization. Further stratifying the associations by season, only in cool season can the statistically significant positive associations be observed. This is somehow unexpected as previous studies in Beijing or the northern part of China found that short-term effects of O_3_ on COPD exacerbation [18] and daily mortality [31] were more pronounced in warm season due to the high ambient concentration, the possible synergistic effects with high temperature as well as the fact that people spend more time outdoors. Previously, Johannson et al. only analysed O_3_ concentrations in summer months and reported a significant positive association with acute exacerbation of IPF [5] whilst Sesé et al. also reported a significant positive association, but without stratifying the risk by season [9]. The studies in Chile [10] and Japan [11] did not report an association with O_3_. As ours is the only study to date that reported seasonal variations of O_3_ associations with IPF hospitalization, these patterns remain to be determined in future studies. An alternative explanation is that O_3_ exposure in cool season may represent a proxy of other more important factors, such as viral infection, which is an known trigger in worsening IPF that may require hospitalization [4]. Being a strong oxidant, O_3_ is known to have the capability to exacerbate chronic lung conditions including asthma and COPD. In addition, O_3_ can cause breathing difficulty by bronchoconstriction, thereby increasing the risk of hospitalization among individuals with existing respiratory diseases including IPF [32]. Animal and human studies have suggested that O_3_ can reach and accumulate at the lower lungs because of its low water solubility, leading to inflammation and impaired small airways function, and potential hospitalization risk [33, 34].

Previous studies either found no associations between acute exacerbation of IPF and SO_2_ exposure [5], or that the associations appeared to be confounded by PM [10]. Despite a remarkable reduction of SO_2_ concentrations by 70% was recorded in Beijing from 2013 to 2017 [18], we still observed that IPF hospitalization risk was significantly higher following recent periods (i.e. past 30 days) of high ambient SO_2_ concentration. Associations did not appear to be significantly affected by further adjustment of PM_2.5_. These results indicated that continued investment on renewable and clean energy, with a steady reduction of traditional fossil energy consumption, still remain pivotal to SO_2_ abatement in order to mitigate the harmful impacts on respiratory diseases. As with O_3_, patients with chronic respiratory diseases may be sensitive to the effects of SO_2_, which has the capacity to induce oxidative stress, airway inflammation and breathing difficulty [35].

We observed a marginally positive association between concurrent-day NO_2_ exposure and IPF hospitalization in the overall population. Interestingly, we found the same effect size for both moving averages 0–30 and lag0. Only in the men-only sample, this positive association became statistically significant. This finding is partly in line with the Johannson study of 436 (80% of them were men) South Korean patients [5], in which higher exposure to previous six-week average NO_2_ was significantly associated with IPF exacerbation. It should be noted that, association with the most acute exposure period (i.e. the week before manifested IPF exacerbation) was weaker and not statistically significant in the Johannson study. Similarly, the Japanese study of 152 patients (70% of them were men) reported significant positive associations between prior 30-day average NO_2_ and IPF acute exacerbation. In contrast, the French study did not find any association with NO_2_ [9]. The time-series study in Chile observed robust positive associations between acute increased exposure to NO_2_ in the previous four days and IPF hospitalization in all populations, with the association being stronger among women [10]. NO_2_ has been commonly studied as an indicator of traffic emission and is usually highly correlated with PM_2.5_, therefore it remains difficult to study its independent effects. Nonetheless, some have argued that NO_2_, via the airway inflammation pathway, could have direct and adverse impacts on respiratory health [36].

We observed that the risk of O_3_ on IPF hospitalization was more pronounced in women, in line with studies which generally found women had a higher respiratory hospitalization risk from O_3_ exposure, particularly during the cold season [33]. And for NO_2_ effects, we only found significant associations in men. The sex-specific (biological factors, e.g. sex hormones, airway diameter, lung size) and gender-specific (socioeconomic and cultural factors, e.g. occupation, personal activity) differences of air pollution effects on respiratory health outcomes still largely remain inconsistent across studies [33], and future studies are warranted to gain better insights into these two different pathways.

To our best knowledge, this is by far the first and the largest time-series study that was conducted in a city with high levels of ambient air pollution, using a relatively large number of IPF hospitalizations over a 5-year period from a representative database. The study has limitations. First, it is an ecological study by design, and hence the modelled risk estimates should not be interpreted as predictive of individual hospitalisation probability. Given the time-series study design, we were unable to adjust for important individual-level factors of IPF such as smoking and socioeconomic status. However, the proportion of these risk factors are unlikely to change substantially in the same population which we were investigating over a short period of time. Notably, time-varying factors such as seasonal viral or bacterial infection will likely have a more important role to confound these relationships, but we did not have these data for further investigation. Second, the use of daily, outdoor, city-wide average air pollutant concentrations inevitably will have introduced bias to health estimates because we cannot take into account place of residence and time-activity patterns (e.g. workplace exposure, travel, indoor cooking) from the study population. Due to privacy reasons, we were only provided area-level (urban district or township) residential address for each patient for this secondary analysis, therefore we cannot model air pollution exposure at individual residence. However, this time-series analysis is an important first step to inform our next immediate work to study the impact of modelled air pollution at residence on health outcomes in a cohort of IPF patients should consent of accessing address data was granted. In addition, personal monitoring of air pollution exposure among IPF patients will provide even more novel insights into the exposure patterns and the associated health risks. Third, as with the Chilean study [10], our outcome was a primary diagnosis of IPF (ICD-10 J84.1) at discharge, which was coded by clinicians and usually the main reason for admission or length of stay. Most IPF-related hospitalizations under J84.1 would be likely related to pneumonia or acute exacerbation, and it is true that neither of which typically manifest clinically within days of a triggering exposure. Therefore, cautious interpretation is needed on our observed significant positive associations in a relative short lag of higher air pollution exposure. Fourth, models including more than two pollutants were not possible due to the high correlation among pollutants. Also, we lacked the information on the occupational history of these patients. Lifetime occupational exposure might have altered the air pollution risk on respiratory health [7] and this is particularly relevant considering the rapid industrialization in Beijing in the last few decades. As pointed out already in the Chilean study, these time-series analyses were mainly to determine whether there was an association between air pollution and acute worsening of IPF that severe enough for hospitalisation. We, however, were unable to determine whether air pollution is the primary cause, or aggravates other intermediate disorders, that ultimately leads to rapid deterioration in IPF patients [10].

## Conclusions

The implementation of Clean Air Action Plan in Beijing successfully reduced the annual mean concentration of PM_2.5_ by one-third (from 87 μg/m^3^ in 2013 to 58 μg/m^3^ in 2017) in the first phase. Although the annual mean PM_2.5_ in Beijing decreased to around 40 μg/m^3^ by end of 2021, it is still eight times higher than the recently updated WHO target value of 5 μg/m^3^, and therefore remains a significant health threat as demonstrated in this study. The concentrations for nitrogen dioxide and ozone, both of which are known to be harmful for respiratory health [31] and the climate, however, remain relatively stable, indicating stringent emission controls are needed, particularly on traffic which is the primary source in Beijing. Tackling these air pollutants should also be recognized as a key player in policies aiming for mitigating impacts from climate change, results of which will also bring health co-benefits.

In conclusion, this study provides evidence that acute exposure to higher levels of air pollution is associated with IPF hospitalization in Beijing. Local air quality policy should be stringently enforced, and carefully monitored for the progress to protect vulnerable populations, the general public and the planet.

## Supplementary Information

**Additional file 1.** **Figure S1.** Air quality monitoring stations across Beijing. **Table S1.** Spearman correlation between air pollutants during 2013-2017. **Table S2.** Associations between PM_2.5_ and PM_10_ (per IQR increase) and IPF hospitalisation in Beijing at different lags during 2013-2017. **Table S3.** Associations between PM_2.5_ (per IQR increase of 83 μg/m^3^) and IPF hospitalisation in Beijing at different lags during 2008-2012. **Figure S2.** Comparisons of associations between PM_2.5_ and IPF hospitalization for periods 2008-2012 and 2013-2017. **Table S4.** Associations between gaseous pollutants (per IQR increase) and IPF hospitalisation in Beijing at different lags during 2013-2017. **Table S5.** Associations between ozone (per IQR increase) and IPF hospitalisation in Beijing at different lags during 2013-2017: by season. **Table S6.** Results of the sensitivity analyses. Table S7 Results of two-pollutant models. **Figure S3.** Exposure-response curves between air pollution exposure and IPF hospitalization at lag0.

## Data Availability

The hospitalisation datasets used and/or analysed for the current study was provided by Beijing Public Health Information Center but restrictions apply to the availability of these data, which were used under license for the current study, and so are not publicly available. Data are however available from the authors upon reasonable request and with permission of Beijing Public Health Information Center. Air quality data are publicly available.

## Abbreviations

CAAP: Clean Air Action Plan
COPD: Chronic obstructive pulmonary disease
FVC: Forced vital capacity
GAM: Generalized additive model
ICD-10: International Classification of Diseases, 10^th^ Revision
ILD: Interstitial lung diseases
IPF: Idiopathic pulmonary fibrosis
IQR: Inter-quartile range
NO_2_: Nitrogen dioxide
O_3_: Ozone
PM: Particulate matter
RR: Relative risk
SO_2_: Sulphur dioxide
WHO: World Health Organisation
