# Supplementary material for: Air pollution and hospitalization of patients with idiopathic pulmonary fibrosis in Beijing: a time-series study
Source: Respir Res. 2022 Apr 5;23:81. doi: 10.1186/s12931-022-01998-8 (PMC8985349; doi:10.1186/s12931-022-01998-8)

**Supplementary files**

**Air pollution and hospitalisation of patients with idiopathic pulmonary fibrosis in Beijing: a time-series study**

Lirong Liang^1,#^, Yutong Cai^2,3#,*^, Baolei Lyu^4^, Di Zhang^1^, Shuilian Chu^1^, Hang Jing^1^, Kazem Rahimi^2,3^, Zhaohui Tong^1,5*^

^1^ Department of Clinical Epidemiology & Tobacco Dependence Treatment Research, Beijing Institute of Respiratory Medicine, Beijing Chaoyang Hospital, Capital Medical University, Beijing, China

^2^Centre for Environmental Health and Sustainability, Department of Health Sciences, Univeristy of Leicester, Leicester, UK

^3^ Nuffield Department of Women’s & Reproductive Health, University of Oxford, Oxford, UK

^4^ Huayun Sounding Meteorology Technology Corporation, Beijing, China

^5^ Department of Respiratory and Critical Care Medicine, Beijing Institute of Respiratory Medicine, Beijing Chaoyang Hospital, Capital Medical University, Beijing, China

^#^ equally contributing

*Correspondence to:

Professor Dr Zhaohui Tong, Department of Respiratory and Critical Care Medicine, Beijing Institute of Respiratory Medicine, Beijing Chaoyang Hospital, Capital Medical University, Beijing 100020, China. Email: [tongzhaohuicy@sina.com](mailto:tongzhaohuicy@sina.com)

Figure E1: Air quality monitoring stations across Beijing


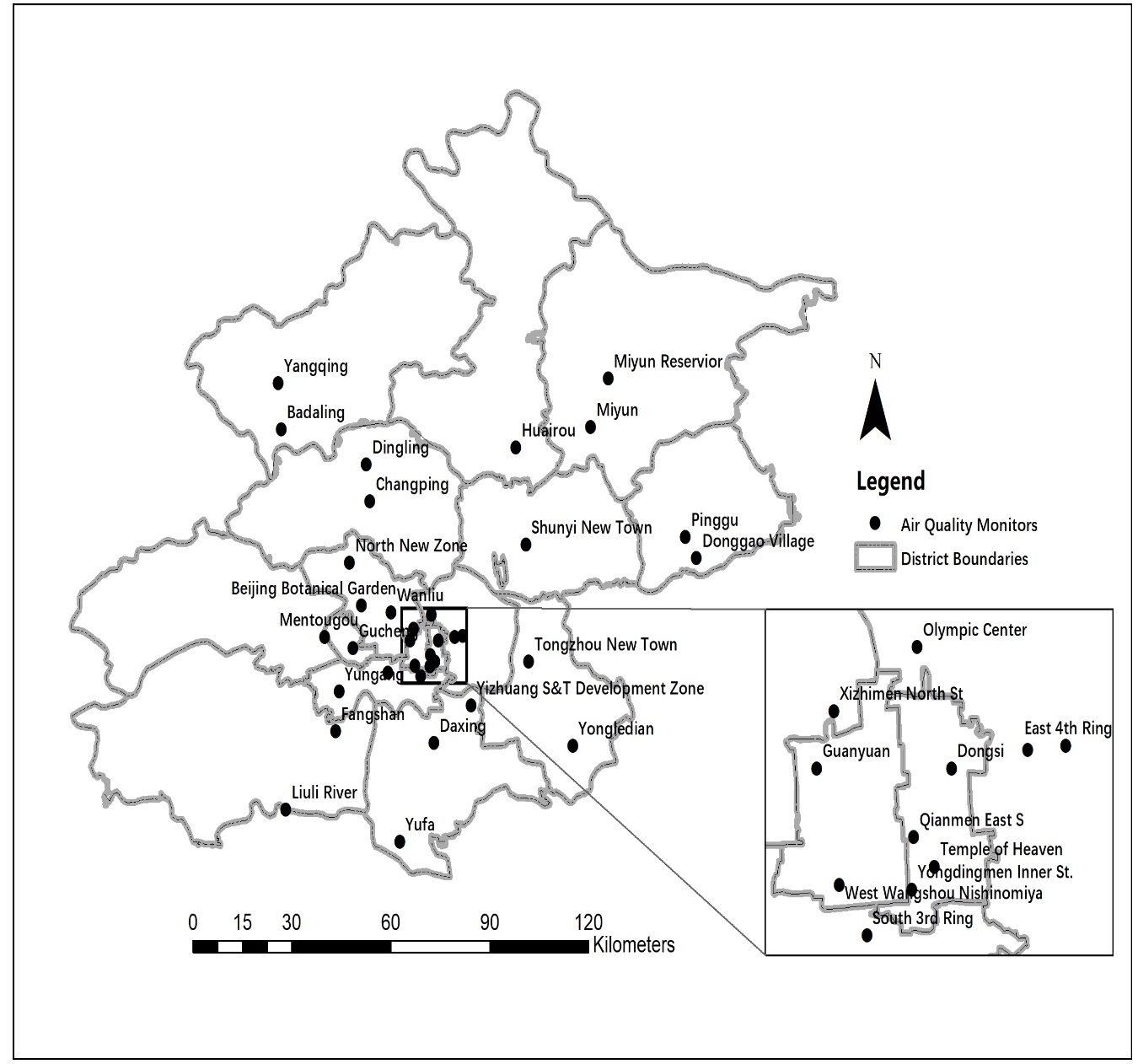


**Table E1** Spearman correlation between air pollutants during 2013-2017

|  | PM_10_ | PM_2.5_ | NO_2_ | SO_2_ | O_3_ |
| --- | --- | --- | --- | --- | --- |
| PM_10_ | - |  |  |  |  |
| PM_2.5_ | 0.93 | - | 0.777 | 0.546 | -0.143 |
| NO_2_ | 0.73 | 0.777 | - | 0.638 | -0.386 |
| SO_2_ | 0.49 | 0.546 | 0.638 | - | -0.289 |
| O_3_ | -0.10 | -0.143 | -0.386 | -0.289 | - |

**Table E2** Associations between PM_2.5_ and PM_10_ (per IQR increase) and IPF hospitalisation in Beijing at different lags during 2013-2017.

|  | **PM_10_** | | | **PM_2.5_** | | |
| --- | --- | --- | --- | --- | --- | --- |
| **lag** | **RR** | **95%CI** | | **RR** | **95%CI** | |
| lag0 | 1.042 | 1.019 | 1.064 | 1.049 | 1.024 | 1.074 |
| lag1 | 1.005 | 0.984 | 1.027 | 1.008 | 0.986 | 1.030 |
| lag2 | 0.998 | 0.978 | 1.019 | 0.998 | 0.977 | 1.019 |
| lag3 | 0.994 | 0.973 | 1.014 | 0.991 | 0.971 | 1.012 |
| lag4 | 1.001 | 0.981 | 1.022 | 0.995 | 0.975 | 1.016 |
|  |  |  |  |  |  |  |
|  | **RR** | **95%CI** | | **RR** | **95%CI** | |
| lag0-1 | 1.027 | 1.005 | 1.050 | 1.031 | 1.007 | 1.056 |
| lag0-2 | 1.019 | 0.997 | 1.042 | 1.021 | 0.998 | 1.044 |
| lag0-3 | 1.012 | 0.991 | 1.034 | 1.013 | 0.990 | 1.036 |
| lag0-4 | 1.011 | 0.989 | 1.033 | 1.009 | 0.987 | 1.032 |
| lag0-30 | 1.021 | 0.994 | 1.049 | 0.994 | 0.962 | 1.027 |

IQR for PM_2.5_ and PM_10_ during 2013-2017 was 72 and 86 μg/m^3^ respectively.

Table E3 Associations between PM_2.5_ (per IQR increase of 83 μg/m^3^) and IPF hospitalisation in Beijing at different lags during 2008-2012.

|  | **lag** | **RR** | **95%CI** |  |
| --- | --- | --- | --- | --- |
| PM_2.5_ | lag0 | 1.062 | 1.025 | 1.101 |
|  | lag1 | 1.035 | 1.002 | 1.070 |
|  | lag2 | 1.031 | 0.999 | 1.065 |
|  | lag3 | 1.020 | 0.988 | 1.054 |
|  | lag4 | 0.988 | 0.956 | 1.020 |
| PM_2.5_ |  |  |  |  |
|  | lag0-1 | 1.054 | 1.020 | 1.090 |
|  | lag0-2 | 1.056 | 1.021 | 1.093 |
|  | lag0-3 | 1.052 | 1.018 | 1.087 |
|  | lag0-4 | 1.041 | 1.007 | 1.076 |
|  |  |  |  |  |

Figure E2 Comparisons of associations between PM_2.5_ and IPF hospitalization for periods 2008-2012 and 2013-2017.


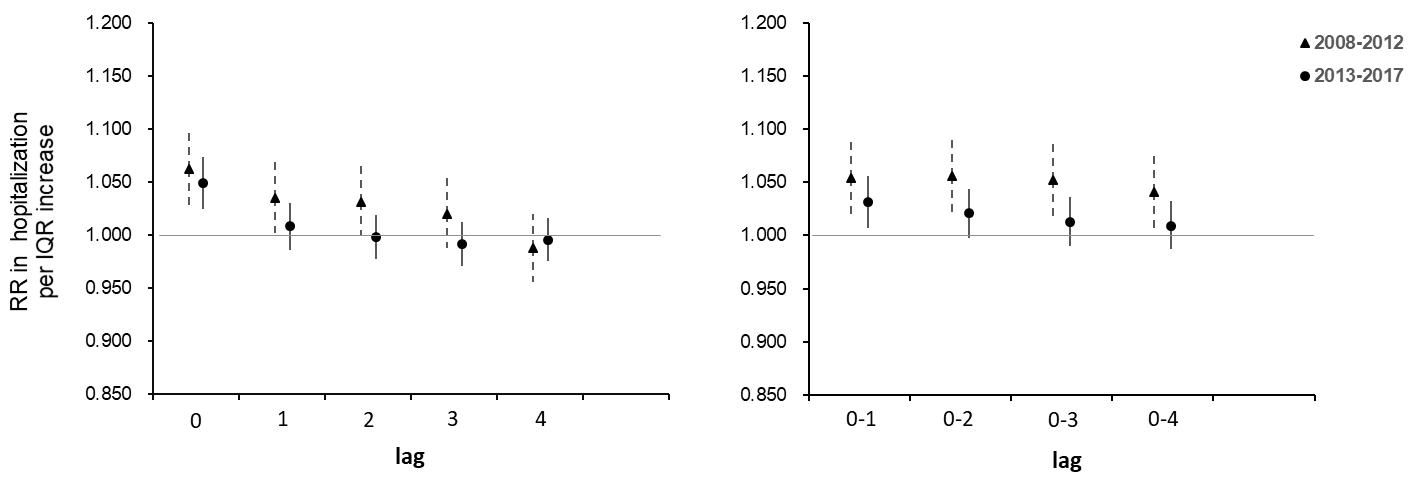


**Table E4** Associations between gaseous pollutants (per IQR increase) and IPF hospitalisation in Beijing at different lags during 2013-2017.

|  | **lag** | **RR** | **95%CI** | |
| --- | --- | --- | --- | --- |
| NO2 | 0 | 1.029 | 0.999 | 1.060 |
| no2_lag1 | 1 | 0.998 | 0.971 | 1.025 |
| no2_lag2 | 2 | 0.983 | 0.958 | 1.009 |
| no2_lag3 | 3 | 0.990 | 0.965 | 1.016 |
| no2_lag4 | 4 | 0.990 | 0.965 | 1.015 |
| SO2 | 0 | 1.023 | 1.002 | 1.045 |
| so2_lag1 | 1 | 1.007 | 0.988 | 1.026 |
| so2_lag2 | 2 | 1.015 | 0.995 | 1.034 |
| so2_lag3 | 3 | 1.017 | 0.997 | 1.037 |
| so2_lag4 | 4 | 1.012 | 0.992 | 1.032 |
| O3 | 0 | 1.045 | 1.000 | 1.092 |
| O3_lag1 | 1 | 1.056 | 1.014 | 1.100 |
| O3_lag2 | 2 | 1.057 | 1.017 | 1.098 |
| O3_lag3 | 3 | 1.037 | 0.998 | 1.077 |
| O3_lag4 | 4 | 1.007 | 0.970 | 1.046 |
| **NO2** |  | **RR** | **95%CI** | |
| no2_lag0-1 | 0-1 | 1.014 | 0.986 | 1.044 |
| no2_lag0-2 | 0-2 | 1.002 | 0.974 | 1.030 |
| no2_lag0-3 | 0-3 | 0.998 | 0.969 | 1.027 |
| no2_lag0-4 | 0-4 | 0.994 | 0.965 | 1.024 |
| no2_lag0-30 | 0-30 | 1.029 | 0.999 | 1.060 |
| **SO2** |  |  |  |  |
| so2_lag0-1 | 0-1 | 1.019 | 0.996 | 1.043 |
| so2_lag0-2 | 0-2 | 1.023 | 0.998 | 1.048 |
| so2_lag0-3 | 0-3 | 1.028 | 1.002 | 1.056 |
| so2_lag0-4 | 0-4 | 1.031 | 1.002 | 1.061 |
| so2_lag0-30 | 0-30 | 1.060 | 1.025 | 1.097 |
| **O3** |  |  |  |  |
| O3_lag0-1 | 0-1 | 1.071 | 1.019 | 1.126 |
| O3_lag0-2 | 0-2 | 1.091 | 1.034 | 1.151 |
| O3_lag0-3 | 0-3 | 1.099 | 1.038 | 1.164 |
| O3_lag0-4 | 0-4 | 1.093 | 1.028 | 1.162 |
| O3_lag0-30 | 0-30 | 1.047 | 0.941 | 1.165 |

**Table E5** Associations between ozone (per IQR increase) and IPF hospitalisation in Beijing at different lags during 2013-2017: by season

|  | RR | 95%CI | |
| --- | --- | --- | --- |
| O_3__warm | 1.035 | 0.977 | 1.097 |
| O_3__warm_lag1 | 1.032 | 0.979 | 1.089 |
| O_3__warm_lag2 | 1.039 | 0.989 | 1.092 |
| O_3__warm_lag3 | 1.026 | 0.977 | 1.077 |
| O_3__warm_lag4 | 0.991 | 0.944 | 1.039 |
| O_3__cold | 1.037 | 0.972 | 1.107 |
| O_3__cold_lag1 | 1.087 | 1.027 | 1.151 |
| O_3__cold_lag2 | 1.086 | 1.030 | 1.144 |
| O_3__cold_lag3 | 1.044 | 0.993 | 1.097 |
| O_3__cold_lag4 | 1.030 | 0.979 | 1.083 |
| O_3__warm_lag0-1 | 1.040 | 0.983 | 1.100 |
| O_3__warm_lag0-2 | 1.048 | 0.993 | 1.106 |
| O_3__warm_lag0-3 | 1.050 | 0.994 | 1.109 |
| O_3__warm_lag0-4 | 1.038 | 0.984 | 1.096 |
| O_3__cold_lag0-1 | 1.089 | 1.017 | 1.166 |
| O_3__cold_lag0-2 | 1.124 | 1.046 | 1.207 |
| O_3__cold_lag0-3 | 1.123 | 1.043 | 1.209 |
| O_3__cold_lag0-4 | 1.118 | 1.037 | 1.205 |

**Table E6** Results of the sensitivity analyses

|  | **PM_2.5_ RR (95%CI)** | | | | | | | | | | |
| --- | --- | --- | --- | --- | --- | --- | --- | --- | --- | --- | --- |
|  |  |  |  |  |  |  |  |  |  |  |  |
|  |  | **Lag0** | **Lag1** | **Lag2** | **Lag3** | **Lag4** | **Lag5** | **Lag0-1** | **Lag0-2** | **Lag0-3** | **Lag0-4** |
| **1** | **2013-2017** | 1.039 (1.015,1.063) | 1.003 (0.982,1.025) | 0.994 (0.974,1.014) | 0.988 (0.968,1.008) | 0.991 (0.971,1.011) | 1.001 (0.982,1.022) | 1.023 (1.000,1.046) | 1.013 (0.991,1.035) | 1.006 (0.984,1.027) | 1.002 (0.981,1.023) |
| **2** | **2013-2017** | 1.047 (1.008,1.088) | 0.990 (0.967,1.014) | 0.989 (0.969,1.010) | 0.986 (0.966,1.006) | 0.990 (0.970,1.010) | 1.001 (0.981,1.021) | 1.008 (0.976,1.040) | 0.997 (0.970,1.024) | 0.991 (0.967,1.016) | 0.989 (0.966,1.012) |
| **3** | **2013-2017** | 1.048 (1.023,1.074) | 1.007 (0.985,1.030) | 0.997 (0.976,1.018) | 0.991 (0.970,1.012) | 0.994 (0.974,1.016) | 1.005 (0.985,1.026) | 1.031 (1.007,1.056) | 1.020 (0.997,1.044) | 1.012 (0.989,1.036) | 1.008 (0.985,1.032) |
| **4** | **2013-2017** | 1.030 (1.008,1.052) | 1.016 (0.994,1.039) | 1.011 (0.989,1.034) | 1.004 (0.982,1.026) | 1.006 (0.984,1.028) | 1.012 (0.991,1.033) | 1.028 (1.005,1.051) | 1.027 (1.004,1.051) | 1.025 (1.001,1.050) | 1.025 (1.000,1.051) |
| **5** | **2013-2017** | 1.020 (0.998,1.041) | 1.006 (0.985,1.029) | 1.002 (0.980,1.024) | 0.995 (0.974,1.017) | 0.999 (0.977,1.020) | 1.014 (0.993,1.036) | 1.015 (0.993,1.037) | 1.012 (0.990,1.035) | 1.009 (0.986,1.031) | 1.007 (0.985,1.030) |

1. = **remove ‘calendar time’**
2. = **replace ‘calendar time’ with an interaction term of exposure-by-season**
3. = **increase the degrees of freedom of temperature and humidity to six**
4. = **model moving averages for lag0–4 of temperature and humidity instead of concurrent day (lag0)**
5. = **model moving averages for lag0–15 of temperature and humidity instead of concurrent day (lag0)**

|  | **RR (95%CI)** | | | | | | | | | | |
| --- | --- | --- | --- | --- | --- | --- | --- | --- | --- | --- | --- |
|  |  | **Lag0** | **Lag1** | **Lag2** | **Lag3** | **Lag4** | **Lag5** | **Lag0-1** | **Lag0-2** | **Lag0-3** | **Lag0-4** |
| **1** | **NO_2_** | 1.021 (0.992,1.050) | 0.994 (0.969,1.020) | 0.980 (0.956,1.005) | 0.987 (0.962,1.012) | 0.986 (0.961,1.011) | 0.988 (0.964,1.012) | 1.008 (0.981,1.035) | 0.996 (0.970,1.023) | 0.992 (0.965,1.020) | 0.988 (0.961,1.016) |
|  | **SO_2_** | 1.011 (0.994,1.029) | 1.001 (0.985,1.018) | 1.007 (0.990,1.024) | 1.009 (0.992,1.026) | 1.005 (0.988,1.022) | 1.001 (0.985,1.019) | 1.008 (0.989,1.027) | 1.009 (0.989,1.029) | 1.011 (0.990,1.032) | 1.011 (0.989,1.034) |
|  | **O_3__warm** | 1.050 (0.994,1.109) | 1.044 (0.992,1.098) | 1.046 (0.998,1.098) | 1.034 (0.986,1.084) | 1.000 (0.955,1.048) | 1.013 (0.968,1.061) | 1.054 (1.000,1.111) | 1.058 (1.007,1.113) | 1.060 (1.008,1.115) | 1.050 (0.999,1.103) |
|  | **O_3__cold** | 1.021 (0.964,1.083) | 1.068 (1.012,1.126) | 1.069 (1.018,1.124) | 1.034 (0.987,1.085) | 1.019 (0.971,1.068) | 1.048 (0.997,1.102) | 1.059 (0.997,1.125) | 1.084 (1.018,1.155) | 1.084 (1.016,1.157) | 1.077 (1.008,1.151) |
| **2** | **NO_2_** | 1.025 (0.975,1.078) | 0.984 (0.956,1.012) | 0.977 (0.953,1.002) | 0.985 (0.961,1.010) | 0.985 (0.961,1.010) | 0.987 (0.963,1.012) | 0.991 (0.954,1.030) | 0.980 (0.948,1.012) | 0.978 (0.948,1.009) | 0.976 (0.946,1.006) |
|  | **SO_2_** | 1.042 (0.994,1.092) | 0.994 (0.973,1.016) | 1.004 (0.985,1.023) | 1.007 (0.988,1.025) | 1.003 (0.985,1.021) | 0.999 (0.980,1.017) | 1.003 (0.967,1.041) | 1.006 (0.975,1.038) | 1.010 (0.979,1.041) | 1.009 (0.978,1.041) |
| **3** | **NO_2_** | 1.026 (0.996,1.058) | 0.996 (0.970,1.024) | 0.982 (0.957,1.008) | 0.990 (0.965,1.016) | 0.989 (0.964,1.015) | 0.991 (0.966,1.017) | 1.012 (0.984,1.042) | 1.000 (0.971,1.029) | 0.996 (0.966,1.026) | 0.992 (0.962,1.022) |
|  | **SO_2_** | 1.025 (1.003,1.047) | 1.009 (0.990,1.029) | 1.017 (0.998,1.037) | 1.020 (1.000,1.040) | 1.014 (0.994,1.034) | 1.010 (0.990,1.031) | 1.022 (0.998,1.046) | 1.027 (1.001,1.053) | 1.033 (1.005,1.062) | 1.037 (1.007,1.068) |
|  | **O_3__warm** | 1.039 (0.980,1.101) | 1.032 (0.978,1.089) | 1.040 (0.989,1.094) | 1.028 (0.979,1.079) | 0.993 (0.946,1.042) | 1.004 (0.958,1.053) | 1.042 (0.985,1.103) | 1.050 (0.994,1.109) | 1.053 (0.996,1.113) | 1.041 (0.986,1.099) |
|  | **O_3__cold** | 1.038 (0.973,1.108) | 1.093 (1.031,1.158) | 1.088 (1.032,1.147) | 1.044 (0.992,1.097) | 1.029 (0.979,1.083) | 1.068 (1.012,1.126) | 1.093 (1.021,1.171) | 1.128 (1.049,1.213) | 1.127 (1.046,1.214) | 1.120 (1.038,1.209) |
| **4** | **NO_2_** | 1.010 (0.984,1.037) | 0.999 (0.973,1.026) | 0.989 (0.963,1.017) | 0.997 (0.970,1.025) | 0.995 (0.968,1.022) | 0.994 (0.969,1.020) | 1.006 (0.980,1.032) | 1.000 (0.973,1.028) | 0.999 (0.969,1.029) | 0.997 (0.966,1.029) |
|  | **SO_2_** | 1.014 (0.994,1.035) | 1.008 (0.989,1.028) | 1.019 (0.999,1.039) | 1.021 (1.001,1.041) | 1.015 (0.996,1.035) | 1.010 (0.991,1.030) | 1.015 (0.991,1.039) | 1.022 (0.997,1.048) | 1.030 (1.002,1.059) | 1.035 (1.004,1.066) |
|  | **O_3__warm** | 1.027 (0.975,1.082) | 1.035 (0.980,1.093) | 1.057 (1.001,1.116) | 1.051 (0.997,1.109) | 1.013 (0.962,1.067) | 1.024 (0.974,1.076) | 1.036 (0.982,1.093) | 1.055 (0.998,1.115) | 1.071 (1.009,1.137) | 1.071 (1.007,1.138) |
|  | **O_3__cold** | 1.002 (0.945,1.063) | 1.053 (0.990,1.120) | 1.065 (1.000,1.133) | 1.017 (0.957,1.080) | 0.998 (0.942,1.058) | 1.047 (0.988,1.108) | 1.036 (0.968,1.109) | 1.075 (0.991,1.167) | 1.087 (0.989,1.195) | 1.082 (0.976,1.200) |
| **5** | **NO_2_** | 1.002 (0.976,1.028) | 0.993 (0.968,1.020) | 0.985 (0.959,1.011) | 0.990 (0.964,1.017) | 0.986 (0.960,1.013) | 0.994 (0.969,1.021) | 0.997 (0.972,1.023) | 0.991 (0.965,1.018) | 0.988 (0.960,1.017) | 0.984 (0.954,1.014) |
|  | **SO_2_** | 1.008 (0.987,1.030) | 1.005 (0.984,1.026) | 1.016 (0.995,1.037) | 1.016 (0.995,1.037) | 1.007 (0.987,1.028) | 1.009 (0.988,1.030) | 1.009 (0.984,1.034) | 1.016 (0.989,1.044) | 1.023 (0.993,1.055) | 1.026 (0.993,1.060) |
|  | **O_3__warm** | 1.037 (0.987,1.090) | 1.039 (0.988,1.093) | 1.054 (1.002,1.108) | 1.047 (0.997,1.100) | 1.016 (0.967,1.067) | 1.032 (0.982,1.084) | 1.043 (0.993,1.096) | 1.055 (1.004,1.108) | 1.065 (1.011,1.121) | 1.062 (1.008,1.118) |
|  | **O_3__cold** | 1.014 (0.958,1.074) | 1.055 (0.993,1.120) | 1.065 (1.001,1.133) | 1.015 (0.955,1.079) | 0.993 (0.936,1.054) | 1.035 (0.972,1.102) | 1.045 (0.979,1.116) | 1.081 (1.000,1.169) | 1.091 (0.996,1.195) | 1.086 (0.982,1.201) |

1. = **remove ‘calendar time’**
2. = **replace ‘calendar time’ with an interaction term of exposure-by-season**
3. = **increase the degrees of freedom of temperature and humidity to six**
4. = **model moving averages for lag0–4 of temperature and humidity instead of concurrent day (lag0)**
5. = **model moving averages for lag0–15 of temperature and humidity instead of concurrent day (lag0)**

Table E7 Results of two-pollutant models

| **PM_2.5_**+SO_2_ | Lag0 | Lag1 | Lag2 | Lag3 | Lag4 |
| --- | --- | --- | --- | --- | --- |
|  | 1.048 (1.018, 1.079) | 1.005 (0.978, 1.032) | 0.984 (0.960, 1.009) | 0.975 (0.951, 1.000) | 0.985 (0.961, 1.009) |
|  | Lag0-1 | Lag0-2 | Lag0-3 | Lag0-4 |  |
|  | 1.029 (1.001, 1.059) | 1.013 (0.985, 1.041) | 1.000 (0.974, 1.027) | 0.996 (0.971, 1.023) |  |
| **PM_2.5_** + O_3_ | Lag0 | Lag1 | Lag2 | Lag3 | Lag4 |
|  | 1.047 (1.021, 1.073) | 1.008 (0.986, 1.031) | 1.000 (0.979, 1.021) | 0.994 (0.974, 1.015) | 0.995 (0.974, 1.016) |
|  | Lag0-1 | Lag0-2 | Lag0-3 | Lag0-4 |  |
|  | 1.028 (1.004, 1.053) | 1.021 (0.997, 1.045) | 1.015 (0.992, 1.039) | 1.012 (0.989, 1.035) |  |
| **NO_2_**+SO_2_ | Lag0 | Lag1 | Lag2 | Lag3 | Lag4 |
|  | 1.015 (0.978, 1.052) | 0.986 (0.952, 1.020) | 0.953 (0.922, 0.985) | 0.964 (0.933, 0.996) | 0.970 (0.939, 1.001) |
|  | Lag0-1 | Lag0-2 | Lag0-3 | Lag0-4 |  |
|  | 1.000 (0.965, 1.036) | 0.979 (0.945, 1.014) | 0.972 (0.938, 1.007) | 0.968 (0.934, 1.003) |  |
| **NO_2_**+O_3_ | Lag0 | Lag1 | Lag2 | Lag3 | Lag4 |
|  | 1.032 (1.002, 1.062) | 1.001 (0.975, 1.027) | 0.991 (0.966, 1.017) | 0.997 (0.972, 1.022) | 0.992 (0.967, 1.017) |
|  | Lag0-1 | Lag0-2 | Lag0-3 | Lag0-4 |  |
|  | 1.017 (0.988, 1.046) | 1.008 (0.980, 1.037) | 1.007 (0.978, 1.037) | 1.004 (0.974, 1.035) |  |
| **SO_2_**+PM_2.5_ | Lag0 | Lag1 | Lag2 | Lag3 | Lag4 |
|  | 1.000 (0.975, 1.026) | 1.004 (0.981, 1.028) | 1.023 (1.000, 1.046) | 1.030 (1.006, 1.054) | 1.020 (0.997, 1.043) |
|  | Lag0-1 | Lag0-2 | Lag0-3 | Lag0-4 |  |
|  | 1.003 (0.976, 1.032) | 1.016 (0.986, 1.046) | 1.028 (0.996, 1.061) | 1.034 (1.000, 1.069) |  |
| **SO_2_**+NO_2_ | Lag0 | Lag1 | Lag2 | Lag3 | Lag4 |
|  | 1.017 (0.992, 1.044) | 1.013 (0.989, 1.038) | 1.037 (1.012, 1.063) | 1.034 (1.009, 1.060) | 1.026 (1.002, 1.051) |
|  | Lag0-1 | Lag0-2 | Lag0-3 | Lag0-4 |  |
|  | 1.019 (0.990, 1.049) | 1.034 (1.003, 1.066) | 1.044 (1.011, 1.078) | 1.049 (1.014, 1.085) |  |
| **SO_2_**+O_3_ | Lag0 | Lag1 | Lag2 | Lag3 | Lag4 |
|  | 1.024 (1.004, 1.044) | 1.007 (0.989, 1.025) | 1.017 (0.999, 1.036) | 1.018 (0.999, 1.037) | 1.013 (0.994, 1.033) |
|  | Lag0-1 | Lag0-2 | Lag0-3 | Lag0-4 |  |
|  | 1.019 (0.996, 1.043) | 1.024 (0.999, 1.049) | 1.028 (1.002, 1.056) | 1.032 (1.002, 1.061) |  |

Figure E3 Exposure-response curves between air pollution exposure and IPF hospitalization at lag0


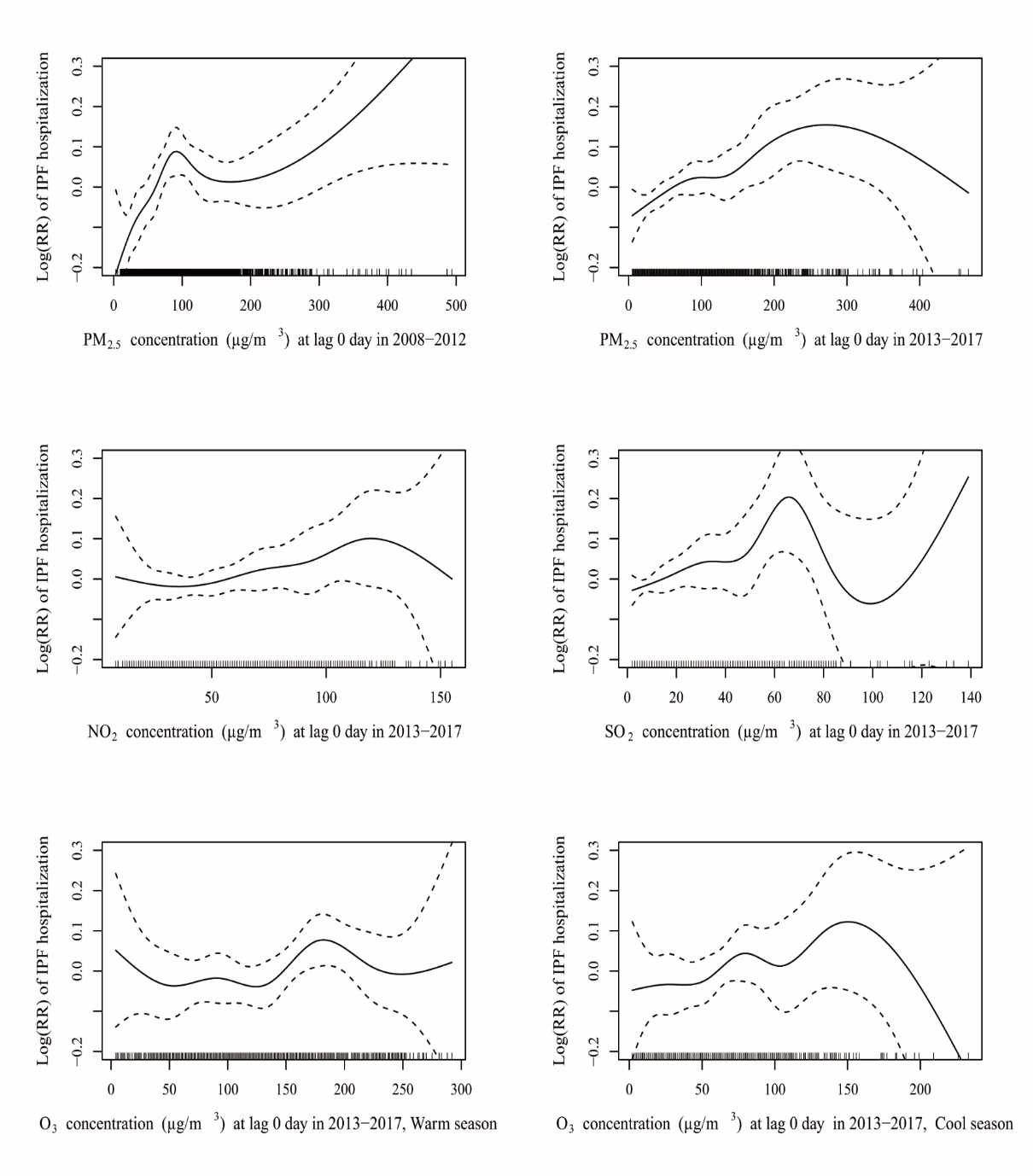

Supplement: Supplementary file 1 — Additional file 1. Figure S1. Air quality monitoring stations across Beijing. Table S1. Spearman correlation between air pollutants during 2013-2017. Table S2. Associations between PM2.5 and PM10 (per IQR increase) and IPF hospitalisation in Beijing at different lags during 2013-2017. Table S3. Associations between PM2.5 (per IQR increase of 83 μg/m3) and IPF hospitalisation in Beijing at different lags during 2008-2012. Figure S2. Comparisons of associations between PM2.5 and IPF hospitalization for periods 2008-2012 and 2013-2017. Table S4. Associations between gaseous pollutants (per IQR increase) and IPF hospitalisation in Beijing at different lags during 2013-2017. Table S5. Associations between ozone (per IQR increase) and IPF hospitalisation in Beijing at different lags during 2013-2017: by season. Table S6. Results of the sensitivity analyses. Table S7 Results of two-pollutant models. Figure S3. Exposure-response curves between air pollution exposure and IPF hospitalization at lag0. [file 12931_2022_1998_MOESM1_ESM.docx]
